# Moderating Role of Multiple Screen Addiction on High Body Mass

**DOI:** 10.1002/fsn3.70383

**Published:** 2025-06-10

**Authors:** Burak Mete, Hakan Demirhindi, Tuba Makca

**Affiliations:** ^1^ Department of Public Health Çukurova Universitesi Adana Türkiye

**Keywords:** hedonia, obesity, screen addiction

## Abstract

Today, the increasing use of multiple media has led to an increase in the concern of screen addiction. This study aimed to evaluate the direct and indirect dimensions of the relationship between multiple screen addiction and body mass. This cross‐sectional study was conducted on 728 university students. The Multiple Screen Addiction Scale, the Hedonistic Eating Scale, and the International Physical Activity Questionnaire were used to measure screen addiction and its effects. Direct and indirect factors were analyzed to evaluate the relationship between screen addiction and body mass index (BMI). The mean age of the students included in the study was 20.78± 3.94 years (min. 18—max. 54). The prevalence of multiple screen addiction was 13%. It was found that hedonic eating scale scores were higher in people with multiscreen addiction. While there was a weak positive correlation between the multiscreen addiction score and hedonistic eating, no significant correlation was found between BMI and physical activity level. Logistic regression analysis showed that the direct effect of multiscreen addiction on BMI was not significant. Multiscreen addiction was an important moderator in the relationship between hedonistic eating and BMI; increasing the mean of multiscreen addiction by one standard deviation led to an increase of 0.073 units in BMI. The moderator effect of multiscreen addiction was significant at high addiction scores. The direct and moderator effects of physical activity on BMI were not found to be significant. High levels of multiple screen addiction are an important moderator in the relationship between high BMI and hedonistic eating. Elucidating the relationship between screen addiction and hedonia will help solve the problem.

## Graphical abstract

High levels of multiple screen addiction strengthen the association between hedonic eating and higher BMI.

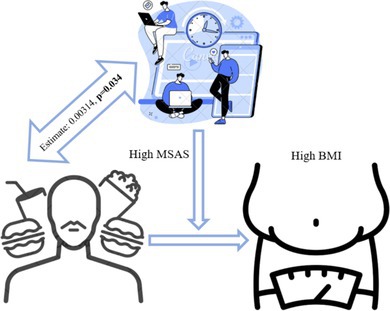

## 1 Introduction

Information and communication technology devices such as phones, tablets, computers, and televisions have become integral to our daily lives (Lin et al. 2020). An increasing number of people around the world participate in the consumption of multiple screen media and use these media tools to perform daily tasks. The fact that devices have more than one function (multitasking) and the emergence of various mobile activities have increased the dependence of individuals on mobile devices (Carrier et al. 2015; Kononova et al. 2014; Lin 2013). Among research on media addiction, increasingly more research is standing out on addiction in general and mobile addiction. According to some authors, media addiction is accepted as a type of behavioral addiction in the absence of external chemical substances' influence. *Screen addiction* is defined as a continuation of unregulated media behaviors using multiple screen devices, and it ranges from compulsive media consumption to highly problematic and even pathological behaviors (LaRose et al. 2003; Lin et al. 2015; Shambare et al. 2012). While media multitasking behavior is regarded as a critical skill for work, education, or entertainment in the digital society of today, on the other hand, very intense screen exposure leads to a decrease in cognitive control and to concerns about socio‐emotional regulation abilities (Pourrazavi et al. 2014; Sahın et al. 2013). Studies on this subject have mostly emphasized the determinants of the types of screen addiction and their effects on the physical, psychological, and socio‐emotional health of individuals (Tokunaga and Rains 2010). Excessive and obsessive media consumption with more than one “screen device” is defined as *multiple screen addiction* (MSA) (Balhara et al. 2018; Bölükbaşı Macit and Kavafoğlu 2019). The main difference between smartphone addiction or internet addiction and MSA is that it does not refer to a situation limited to a single tool or service, and the individual feels discomfort and deprivation in case of simultaneous loss of access to all or some of these devices. Feeling discomfort in the absence or restriction of access to an object or situation is one of the important indicators of behavioral addiction (Lin et al. 2020). The 2020 Digital Report revealed that internet users aged between 16 and 64 in Türkiye watched TV for an average of 3 h, used the internet for 7.5 h, and played console games for 1 h every day (Kemp, n.d.). Screen exposure appears as an important dimension in the adult population. Increased screen time due to MSA leads to decreased physical activity and a sedentary lifestyle, often accompanied by mindless eating. Whether MSA leads to overweight or obesity is still in discussion. The mechanism, the moderators or mediators, if any, of the relationship between MSA and weight gain still needs to be elucidated (Coombs and Stamatakis 2015; Haghjoo et al. 2022; Wansink 2010). We aimed to investigate the relationship between MSA and body mass index (BMI), hedonic eating habits, and physical activity in university students.

## 2 Materials and Methods

### 2.1 Research Type and Ethics

This cross‐sectional study was conducted in 2024 in the Department of Public Health of the _xxx_ University's Faculty of Medicine, _city_, _country_. It included the university students of the Vocational School of Health Sciences and the Faculty of Medicine.

Ethical approval was obtained from the Non‐Interventional Clinical Research Ethics Committee of the Faculty of Medicine in _xxx_ University (Dated 04.01.2024, Meeting No. 140, Decree No. 30). Consents were requested from participants for the data used in the study. The questionnaire forms were sent to the students via their social media accounts as an online survey.

### 2.2 Determination of Sample Size and Selection of Participants

The minimum sample size to be reached in the study was calculated as 616, assuming a Type 1 error of 0.05, a power of 0.8, and an effect size of 0.1 in a one‐tailed test. The sample size was increased by approximately 15% due to possible nonresponse, and 728 people were reached. The convenience sampling method was used in the sampling. An informed consent form was signed by the participants, and only those who participated voluntarily were included in the study. The sampling was terminated when the determined sample size was reached.

### 2.3 Measuring Instruments

#### 2.3.1 Multiple Screen Addiction Scale (MSAS)

This scale was developed by Sarıtepeci M in 2021, and its validity and reliability were also determined by the same author (Sarıtepeci 2021). The scale is a 5‐point Likert type. Each item in the scale is rated by the participants between “1 (never)” and “5 (always).” The total score obtained from the scale is used to assess addiction status, that is, the higher the score obtained from the scale, the higher the risk of MSA. The evaluation is based on “monothetic” and/or “polythetic” criteria. Monothetic criteria require participants to score above a certain threshold for each item in the scale to be categorized as “addict;” that is, in the 15‐item MSA scale, a participant must score ≥ 3 on all 15 items to be classified as “addict.” In the case of polythetic criteria, the assessment is based on scoring high for a subset of the scale, for example, in half of the items. In the MSA scale, a participant will be classified as an “addict” if he/she scores ≥ 3 in at least eight of the 15 items (Sarıtepeci 2021).

#### 2.3.2 Hedonistic Eating Scale (HES)

The hedonistic eating scale was developed by Atik et al. in 2019. Content validity, factor analysis, and item analysis gave rise to a single‐component (unidimensional) 15‐item scale. The internal consistency (Cronbach's alpha coefficient) of the scale is 0.968. All items are scored as 1, 2, 3, 4, or 5, with a final score between 15 and 75. The score obtained is directly proportional to the level of hedonic eating habits (Atik et al. 2019).

#### 2.3.3 International Physical Activity Questionnaire (IPAQ)—Short Form

The International Physical Activity Questionnaire—Short Form was used to determine participants' physical activity level. The IPAQ, consisting of seven questions, applies to people between 15 and 69 years of age and has five domains: job‐related physical activity, transportation physical activity, housework and house maintenance physical activity, recreation or leisure‐time physical activity, and time spent sitting. The IPAQ demonstrates acceptable test–retest reliability and concurrent and criterion validity in an international study conducted across 12 countries (Craig et al. 2003). The Turkish validity and reliability study was performed by Öztürk M. in 2005 (Öztürk 2005). Scoring for the IPAQ is expressed in metabolic minutes (MET‐min) per week of physical activity. One MET is the energy a person expends while at rest. Accordingly, energy expended in other activities is calculated as a multiple of this resting reference value and is obtained using standardized multipliers, which are 3.3 for walking, 4.0 for moderate intensity, and 8.0 for vigorous‐intensity activities. The total score calculation can be described as follows:

IPAQ score = Standardized multiplier× duration (minutes) × frequency (days per week) of an activity.

Bouts of activity lasting less than 10 min are not counted, while those of more than 3 h are truncated to 180 min. The sum of MET‐min/week of all the activities performed during the week will give the total IPAQ score in MET‐min. For example, if we assume that a person walked 4 h per day for 5 days a week, the weekly MET‐min for walking activity will be: Walking MET‐min/week = 3.3 × 180 × 5 = 2970 MET‐min, where 3.3 is the standardized multiplier for walking, and 4 h (240 min) are truncated to 180 min.

Another form of output from scoring the IPAQ is the “categorical” output, which is reported as low, moderate, or high activity levels. Scoring a high level of physical activity on the IPAQ means a person's physical activity level equates to approximately 1 h of activity per day or more at least a moderate‐intensity activity level. Scoring a moderate level of physical activity on the IPAQ means a person is doing some activity more than likely equivalent to half an hour of at least moderate‐intensity physical activity on most days. Scoring a low level of physical activity on the IPAQ means that a person is not meeting any of the criteria for either moderate or high levels of physical activity (Forde 2025).

## 3 Results

The mean age of 728 individuals included in our study was 20.78 ± 3.94 (min. 18—max. 54). Based on the MSAS, the participants were categorized into two groups: “addicts” and “nonaddicts.” The MSA rate was 13%, with 95 participants falling into the “addicts” group. When the participant characteristics were compared between MSA groups, average daily screen time and HES scores were found to be statistically significantly higher in the “addicts” group, while no difference was observed for age, sex, physical activity level, and BMI between addiction groups (Table 1).

**TABLE 1:** Comparison of the participant characteristics and scale scores between the multiple screen addiction groups.

Variables	MSAS groups		*p*

Addicts *n* = 95 (13%)	Nonaddicts *n* = 633 (87%)	
Age	20.38± 1.99	20.84 ± 4.15	0.293
Male/Female ratio (*n* (%))	62 (65.3)/33 (34.7)	430 (67.9)/203 (32.1)	0.605
Body mass index	22.90 ± 4.18	22.92 ± 4.06	0.960
Screen time (min)	306.0 ± 142.64	264.22 ± 135.68	**0.006**
HES score	51.76 ± 11.71	44.33 ± 10.61	**< 0.001**
Physical activity (MET‐min)	3078.11 ± 2593.41	3221.07 ± 2644.99	0.623

*Note:* *p*, statistical significance; Significant values are in bold.

Abbreviations: HES, hedonistic eating scale score; MET‐min, metabolic minutes per week based on IPAQ (international physical activity questionnaire) to measure physical activity level; MSAS, multiple screen addiction scale score.

When the correlations between the scale scores and BMI, physical activity level, or age were examined, it was found that the MSAS score showed a weak positive correlation with the HES score and a very weak positive correlation with age (Table 2).

**TABLE 2:** Correlations between scale scores.

		Total MSAS score	HES score	BMI	Physical activity
HES score	r	0.307**			
	*p*	< 0.001			
BMI	r	0.041	0.047		
	*p*	0.266	0.207		
Physical activity	r	0.011	−0.027	0.112**	
	*p*	0.774	0.460	0.003	
Age	r	0.077*	−0.046	0.205**	0.063
	*p*	0.039	0.214	< 0.001	0.089

*Note:* *p*, statistical significance; *significant and very weak correlation; **significant and weak correlation.

Abbreviations: HES, hedonistic eating scale score; MET‐min, metabolic minutes per week based on IPAQ (international physical activity questionnaire) to measure physical activity level; MSAS, multiple screen addiction scale score.

A logistic regression model was constructed to predict the risk of obesity or overweight under the effect of age, sex, HES score, MSAS score, and physical activity. The model was statistically significant (omnibus test *p* < 0.001), and the goodness of fit of the model was adequate (Nagelkerke *R* ^*2*^ = 0.135). The accuracy rate of the model was 74.4%. Among the variables included in the model, the risk of being obese/overweight was found to be increased 1.093 times by each one‐unit increase in age, 3.046 times by being male, and 1.020 times by each one‐unit increase in the HES score (Table 3).

**TABLE 3:** Logistic regression analysis for estimating the risk of being obese and overweight.

					95% C.I. for O.R.	
Variables	B	S.E.	*p*	O.R.	Lower	Upper
Age	0.089	0.023	**< 0.001**	1.093	1.046	1.144
Sex	1.114	0.182	**< 0.001**	3.046	2.132	4.353
HES score	0.020	0.008	**0.021**	1.020	1.003	1.037
MSAS score	0.002	0.008	0.764	1.002	0.987	1.018
Physical activity	0.000	0.000	0.369	1.000	1.000	1.000
Constant	−3.824	0.696	< 0.001	0.022		

*Note:* *p*, statistical significance; Significant values are in bold.

Abbreviations: B, beta; C.I, confidence interval; HES, hedonistic eating scale score; MET‐min, metabolic minutes per week based on IPAQ (International Physical Activity Questionnaire) to measure physical activity level; MSAS, multiple screen addiction scale score; O.R, odds ratio; S.E, standard error for beta.

**FIGURE 1:**
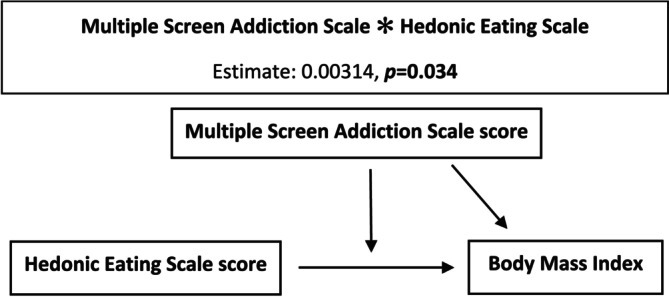
Moderation model 1.

When the moderator effect of MSA on the relationship between hedonic eating and BMI was analyzed, the MSAS score was found to be a significant moderator (*p* = 0.036) (Table 4, Figure 1).

**TABLE 4:** Moderating role of multiscreen addiction on the relationship between hedonic eating and body mass index.

			95% C.I.			
	Estimate	S.E.	Lower	Upper	Z	*p*
HES	0.03618	0.01587	0.00682	0.06923	2.28	0.023
MSAS	−0.01696	0.01367	−0.04520	0.00964	−1.24	0.215
HES ✻ MSAS	0.00314	0.00148	3.69e‐4	0.00631	2.12	0.034

*Note:* *p*, statistical significance.

Abbreviations: C.I, confidence interval; HES, hedonistic eating scale score; MSAS, multiple screen addiction scale score; S.E, standard error.

**FIGURE 2:**
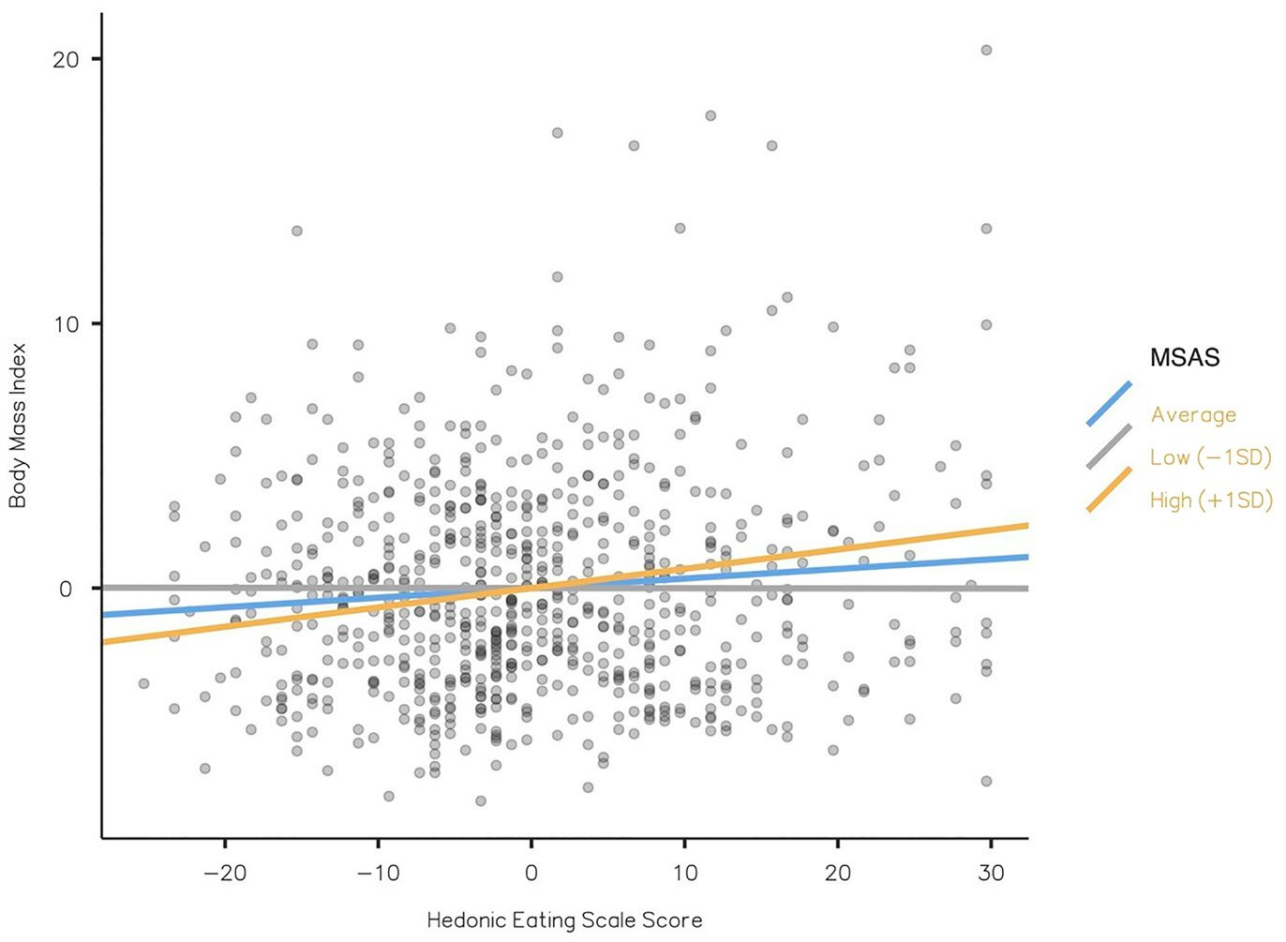
Simple slope plot.

It was found that the moderator effect of high MSAS scores was significant (*p* = 0.002), while low values were not significant (*p* = 0.981). When the mean of the MSAS score was increased by one standard deviation, it was seen that it led to an increase of 0.073 units in BMI. The effect of hedonic eating behavior on BMI increases at high MSAS scores (Table 5, Figure 2).

**TABLE 5:** Simple slope estimates of the moderating role of multiscreen addiction on the relationship between hedonic eating and body mass index.

			95% C.I.			
	Estimate	S.E.	Lower	Upper	Z	*p*
Average	0.0362	0.0158	0.00710	0.0697	2.2909	0.022
Low (−1 SD)	−5.70e‐4	0.0231	−0.04586	0.0473	−0.0247	0.980
High (+1 SD)	0.0730	0.0238	0.02732	0.1220	3.0752	0.002
The effect of the predictor (HES) on the dependent variable (BMI) at different levels of the moderator (MSAS)						

*Note:* *p*, statistical significance.

Abbreviations: BMI, body mass index; C.I, confidence interval; HES, hedonistic eating scale score; MSAS, multiple screen addiction scale score; S.E, standard error.

MSAS, Multiple Screen Addiction Scale score.

**FIGURE 3:**
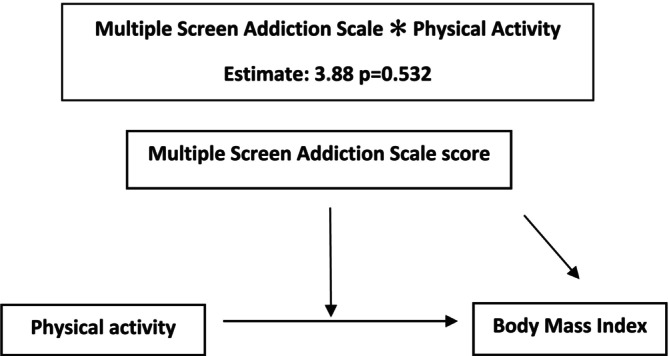
Moderation model 2.

When the moderator effect of MSAS in the relationship between physical activity level and BMI was analyzed, it was found that the MSAS score was not a significant moderator (*p* = 0.532) (Figure 3).

## 4 Discussion

In parallel with today's developments, multimedia use is gradually increasing, and it is regarded as a critical skill in the digital society, but there is a concern that it may be associated with screen addiction (Pourrazavi et al. 2014). In our study, the factors that are important in the relationship between MSA and high BMI were analyzed. We found that HES scores were higher in the group with MSA, and there was a positive correlation between the MSAS and HES scores. MSA was also determined to be an important moderator in the relationship between hedonic eating and BMI, and one standard deviation increase in the mean of the MSAS score resulted in an increase of 0.073 units in BMI. This moderator effect was found to be significant, particularly at high levels of MSAS scores.

In the study conducted by Haghjoo et al., the probability of developing overweight/obesity was found to be 1.27 times higher in adolescents in the highest screen time category. It was reported that there was a positive relationship between screen time and overweight/obesity, but there was no dose–response evidence (Haghjoo et al. 2022). In our study, no significant direct relationship was found between MSAS score, screen exposure time, and the risk of obesity/overweight. Our results indicated no direct relationship between high BMI and screen addiction. Focusing on the indirect or moderating effects of screen addiction would be more useful in elucidating this relationship. In the literature, the hypothesis that screen exposure triggers a sedentary life and leads to inactivity and obesity is a well‐studied subject. Sedentary behaviors are defined as activities performed in a sitting position, such as watching television or other screen activities, and are characterized by low energy expenditure (Coombs and Stamatakis 2015). During and after adolescence, physical activity decreases, getting replaced by increasing inactivity in human life. According to some studies, inactivity associated with increased screen exposure leads to increased obesity (Throuvala et al. 2020). Many studies reported a relationship between screen time and body fat ratio; however, the results were inconsistent. Some studies reported that the probability of obesity increases with increasing screen time (Cheng et al. 2020; Dalamaria et al. 2020; Franceschin and da Veiga 2020), while some denied this relationship (De Lima et al. 2020; Haidar et al. 2019; Kerkadi et al. 2019; Lopez‐Gonzalez et al. 2020; Saha et al. 2018). The findings of our study indicated that hedonistic eating behavior during screen time was effective in having a high BMI. The moderating effect of screen addiction on physical activity and hedonic eating behavior seems to be mostly through hedonic eating. In particular, in the relationship between hedonic eating and obesity, high screen addiction levels were found to be effective moderators. In the study conducted by Aşut et al., eating snacks while watching television was found to be significantly associated with obesity (Aşut et al. 2019). Koca et al. found that BMI showed a positive and significant correlation with internet addiction scores and food addiction scores. Considering the relationship between BMI and addiction, the effect of internet and food addiction on obesity was reported to be significant (Koca et al. 2023). In the study conducted by Bozkurt et al., obese people were found to have higher rates of internet addiction compared to their healthy peers, and the results showed a relationship between internet addiction and BMI (Bozkurt et al. 2018). Kaur showed that exaggerated hedonic responses were associated with higher body mass in adolescents. A hedonic reward, which is a kind of construct associated with tasty foods, might encourage excessive energy intake. These results suggested that hedonic hunger might potentially override homeostatic energy needs and be associated with weight gain (Kaur 2018). Bejarano et al. reported that hedonic hunger was positively associated with fatty and starchy food consumption and controlled diet motivation characterized by the desire to please others, conform to social norms, gain respect, or avoid guilt or shame. People with controlled diet motivation may be vulnerable to the effect of hedonic hunger and may be prone to consume higher amounts of starchy foods and fast food (Bejarano and Cushing 2018).

Obesity is a condition in which genetic, behavioral, and environmental factors play a role together (Patel et al. 2009). In a study by Saper et al., it was defined that eating behavior is regulated by two different systems: homeostatic and hedonic (Saper et al. 2002). According to the hedonistic view, the desire to consume delicious foods increases as a result of pleasure. Therefore, it is important to establish a balanced relationship between lifestyle and food consumption (Lowe and Butryn 2007). Christodoulou et al. found that adolescents may become desensitized as a result of prolonged screen exposure and may show a blunted response to hedonic effects resulting from increased screen time. This increased anhedonia and the need to compensate for reduced reward experience may result in an increased risk of other pleasurable behaviors (Christodoulou et al. 2020). These results provide some clarity to the relationship between high screen addiction and hedonic eating in our results. The anhedonia that develops at the end of prolonged screen exposure may result in unnecessary eating and excessive energy intake. Anhedonia as a result of desensitization and motivation for controlled dieting may lie behind the relationship between MSA and high BMI.

The strength of the study is that it is one of the few studies in the literature that examines the indirect dimensions of the relationship between MSA and obesity. However, some limitations should be considered when interpreting our findings. The fact that the questions were based on declaration may have led to information bias, and the fact that only volunteers answered the questionnaire may have led to selection bias. We tried to minimize the effect of these limitations by increasing the sample size.

## 5 Conclusion

The results of our study showed that MSA had a significant effect as a moderator in having high BMI in young adults. High MSA led to high BMI by increasing hedonic eating. No direct or indirect effect of physical activity was found to be effective. Elucidation of the relationship between screen addiction and hedonia seems to be important in solving the problem. Studies are needed to elucidate the mechanism between screen exposure, duration, and hedonia.

## Author Contributions

**Burak Mete:** conceptualization (equal), formal analysis (equal), methodology (equal), project administration (equal), supervision (equal), writing – review and editing (equal). **Hakan Demirhindi:** conceptualization (equal), methodology (equal), writing – review and editing (equal). **Tuba Makca:** conceptualization (equal), formal analysis (equal), methodology (equal), writing – review and editing (equal).

## Conflicts of Interest

The authors declare no conflicts of interest.

## Data Availability

The data that support the findings of this study are available from the corresponding author upon a reasonable request.

## References

[fsn370383-bib-0001] Aşut, Ö. , G. Abuduxike , S. Acar‐Vaizoğlu , and S. Cali . 2019. “Relationships Between Screen Time, Internet Addiction and Other Lifestyle Behaviors With Obesity Among Secondary School Students in the Turkish Republic of Northern Cyprus.” Turkish Journal of Pediatrics 61, no. 4: 568–579. 10.24953/TURKJPED.2019.04.014. 31990475

[fsn370383-bib-0002] Atik, D. , A. Neşe , and U. Özcan Yüce . 2019. “Scale Development Study: Hedonistic Eating Scale.” Acta Medica Alanya 3, no. 2: 147–153. 10.30565/medalanya.545200.

[fsn370383-bib-0003] Balhara, Y. P. S. , K. Verma , and R. Bhargava . 2018. “Screen Time and Screen Addiction: Beyond Gaming, Social Media and Pornography– A Case Report.” Asian Journal of Psychiatry 35: 77–78. 10.1016/J.AJP.2018.05.020. 29803121

[fsn370383-bib-0004] Bejarano, C. M. , and C. C. Cushing . 2018. “Dietary Motivation and Hedonic Hunger Predict Palatable Food Consumption: An Intensive Longitudinal Study of Adolescents.” Annals of Behavioral Medicine : A Publication of the Society of Behavioral Medicine 52, no. 9: 773–786. 10.1093/ABM/KAX051. 30124763

[fsn370383-bib-0005] Bölükbaşı Macit, Z. , and S. Kavafoğlu . 2019. “Screen: Subject of all Information Technology Addiction.” Middle Black Sea Journal of Health Science 5, no. 3: 293–301. 10.19127/mbsjohs.542122.

[fsn370383-bib-0006] Bozkurt, H. , S. Özer , S. Şahin , and E. Sönmezgöz . 2018. “Internet Use Patterns and Internet Addiction in Children and Adolescents With Obesity.” Pediatric Obesity 13, no. 5: 301–306. 10.1111/ijpo.12216. 28371539

[fsn370383-bib-0007] Carrier, L. M. , L. D. Rosen , N. A. Cheever , and A. F. Lim . 2015. “Causes, Effects, and Practicalities of Everyday Multitasking.” Developmental Review 35: 64–78. 10.1016/j.dr.2014.12.005.

[fsn370383-bib-0008] Cheng, L. , Q. Li , A. Hebestreit , et al. 2020. “The Associations of Specific School‐ and Individual‐Level Characteristics With Obesity Among Primary School Children in Beijing, China.” Public Health Nutrition 23, no. 10: 1838–1845. 10.1017/S1368980019004592. 32279683 PMC10200460

[fsn370383-bib-0009] Christodoulou, G. , A. Majmundar , C. Chou , and M. A. Pentz . 2020. “Anhedonia, Screen Time, and Substance Use in Early Adolescents: A Longitudinal Mediation Analysis.” Journal of Adolescence 78, no. 1: 24–32. 10.1016/j.adolescence.2019.11.007. 31812941 PMC6935414

[fsn370383-bib-0010] Coombs, N. A. , and E. Stamatakis . 2015. “Associations Between Objectively Assessed and Questionnaire‐Based Sedentary Behaviour With BMI‐Defined Obesity Among General Population Children and Adolescents Living in England.” BMJ Open 5, no. 6: e007172. 10.1136/BMJOPEN-2014-007172. PMC4480033 26088807

[fsn370383-bib-0011] Craig, C. L. , A. L. Marshall , M. Sjöström , et al. 2003. “International Physical Activity Questionnaire: 12‐Country Reliability and Validity.” Medicine and Science in Sports and Exercise 35, no. 8: 1381–1395. 10.1249/01.MSS.0000078924.61453.FB. 12900694

[fsn370383-bib-0012] Dalamaria, T. , W. De Jesus Pinto , E. Dos Santos Farias , and De Souza, OF . 2020. “Internet Addiction Among Adolescents in a Western Brazilian Amazonian City.” Revista Paulista de Pediatria 39: e2019270. 10.1590/1984-0462/2021/39/2019270. 32638945 PMC7333942

[fsn370383-bib-0013] De Lima, T. R. , M. S. Moraes , J. H. C. Andrade , J. M. De Farias , and D. A. S. Silva . 2020. “Associated Factors With the Isolated and Simultaneous Presence of Overweight and Abdominal Obesity in Adolescents.” Revista Paulista de Pediatria: Orgao Oficial da Sociedade de Pediatria de Sao Paulo 38: 1–10. 10.1590/1984-0462/2020/38/2018332. PMC7212557 32401945

[fsn370383-bib-0014] Forde, C. 2025. Scoring the International Physical Activity Questionnaire (IPAQ). University of Dublin. https://ugc.futurelearn.com/uploads/files/bc/c5/bcc53b14‐ec1e‐4d90‐88e3‐1568682f32ae/IPAQ_PDF.pdf.

[fsn370383-bib-0015] Franceschin, M. J. , and G. V. da Veiga . 2020. “Association of Cardiorespiratory Fitness, Physical Activity Level, and Sedentary Behavior With Overweight in Adolescents.” Revista Brasileira de Cineantropometria & Desempenho Humano 22: e60449. 10.1590/1980-0037.2020V22E60449.

[fsn370383-bib-0016] Haghjoo, P. , G. Siri , E. Soleimani , M. A. Farhangi , and S. Alesaeidi . 2022. “Screen Time Increases Overweight and Obesity Risk Among Adolescents: A Systematic Review and Dose‐Response Meta‐Analysis.” BMC Primary Care 23, no. 1: 161. 10.1186/S12875-022-01761-4. 35761176 PMC9238177

[fsn370383-bib-0017] Haidar, A. , N. Ranjit , N. Archer , and D. M. Hoelscher . 2019. “Parental and Peer Social Support Is Associated With Healthier Physical Activity Behaviors in Adolescents: A Cross‐Sectional Analysis of Texas School Physical Activity and Nutrition (TX SPAN) Data.” BMC Public Health 19, no. 1: 1–9. 10.1186/S12889-019-7001-0/TABLES/4. 31132999 PMC6537420

[fsn370383-bib-0018] Kaur, K. 2018. “Does Dietary Behavior Mediate the Association Between Hedonic Hunger and BMI in Overweight/Obese Adolescents?” [Utah, U.S.A.]: Brigham Young University. https://scholarsarchive.byu.edu/etd [Accessed 2nd March 2025].

[fsn370383-bib-0019] Kemp, S. n.d. “*Digital 2020: Global Digital Overview — DataReportal – Global Digital Insights.”* https://datareportal.com/reports/digital‐2020‐global‐digital‐overview [Accessed 2nd March 2025].

[fsn370383-bib-0020] Kerkadi, A. , A. H. Sadig , H. Bawadi , et al. 2019. “The Relationship Between Lifestyle Factors and Obesity Indices Among Adolescents in Qatar.” International Journal of Environmental Research and Public Health 16, no. 22: 4428–4443. 10.3390/IJERPH16224428. 31766192 PMC6888352

[fsn370383-bib-0021] Koca, S. B. , A. Paketci , and G. Buyukyilmaz . 2023. “The Relationship Between Internet Usage Style and Internet Addiction and Food Addiction in Obese Children Compared to Healthy Children.” Turkish Archives of Pediatrics 58, no. 2: 205–211. 10.5152/TurkArchPediatr.2023.22183. 36856359 PMC10081003

[fsn370383-bib-0022] Kononova, A. , T. Zasorina , N. Diveeva , A. Kokoeva , and A. Chelokyan . 2014. “Multitasking Goes Global: Multitasking With Traditional and New Electronic Media and Attention to Media Messages Among College Students in Kuwait, Russia, and the USA.” International Communication Gazette 76, no. 8: 617–640. 10.1177/1748048514548533.

[fsn370383-bib-0023] LaRose, R. , C. A. Lin , and M. S. Eastin . 2003. “Unregulated Internet Usage: Addiction, Habit, or Deficient Self‐Regulation?” Media Psychology 5, no. 3: 225–253. 10.1207/S1532785XMEP0503_01.

[fsn370383-bib-0024] Lin, L. 2013. “Multiple Dimensions of Multitasking Phenomenon.” International Journal of Technology and Human Interaction 9, no. 1: 37–49. 10.4018/jthi.2013010103.

[fsn370383-bib-0025] Lin, T. T. C. , Y. H. Chiang , and Q. Jiang . 2015. “Sociable People Beware? Investigating Smartphone Versus Nonsmartphone Dependency Symptoms Among Young Singaporeans.” Social Behavior and Personality: An International Journal 43, no. 7: 1209–1216. 10.2224/sbp.2015.43.7.1209.

[fsn370383-bib-0026] Lin, T. T. C. , A. Kononova , and Y. H. Chiang . 2020. “Screen Addiction and Media Multitasking Among American and Taiwanese Users.” Journal of Computer Information Systems 60, no. 6: 583–592. 10.1080/08874417.2018.1556133.

[fsn370383-bib-0027] Lopez‐Gonzalez, D. , A. Partida‐Gaytán , J. C. Wells , et al. 2020. “Obesogenic Lifestyle and Its Influence on Adiposity in Children and Adolescents, Evidence From Mexico.” Nutrients 12, no. 3: 819. 10.3390/NU12030819. 32204522 PMC7146202

[fsn370383-bib-0028] Lowe, M. R. , and M. L. Butryn . 2007. “Hedonic Hunger: A New Dimension of Appetite?” Physiology & Behavior 91, no. 4: 432–439. 10.1016/j.physbeh.2007.04.006. 17531274

[fsn370383-bib-0029] Öztürk, M. 2005. “A research on reliability and validity of international physical activity questionnaire and determination of physical activity level in university students.” [Master's Degree Thesis] [Ankara]: Hacettepe University, Health Sciences Institute.

[fsn370383-bib-0030] Patel, M. S. , M. Srinivasan , and S. G. Laychock . 2009. “Metabolic Programming: Role of Nutrition in the Immediate Postnatal Life.” Journal of Inherited Metabolic Disease 32, no. 2: 218–228. 10.1007/s10545-008-1033-4. 19096914

[fsn370383-bib-0031] Pourrazavi, S. , H. Allahverdipour , M. A. Jafarabadi , and H. Matlabi . 2014. “A Socio‐Cognitive Inquiry of Excessive Mobile Phone Use.” Asian Journal of Psychiatry 10: 84–89. 10.1016/j.ajp.2014.02.009. 25042958

[fsn370383-bib-0032] Saha, M. , D. Krishna Adhikary , I. Parvin , Y. Raj Sharma , F. Akhter , and M. Majumder . 2018. “Obesity and Its Risk Factors of Among School Children in Sylhet, BangladeshIssue 39 Apr‐J Nepal Health Res Counc.” Journal of Nepal Health Research Council 16, no. 2: 205–213. 10.3126/jnhrc.v16i2.20311. 29983438

[fsn370383-bib-0033] Sahın, S. , K. Ozdemir , A. Unsal , and N. Temiz . 2013. “Evaluation of Mobile Phone Addiction Level and Sleep Quality in University Students.” Pakistan Journal of Medical Sciences 29, no. 4: 913–918. 10.12669/pjms.294.3686. 24353658 PMC3817775

[fsn370383-bib-0034] Saper, C. B. , T. C. Chou , and J. K. Elmquist . 2002. “The Need to Feed: Homeostatic and Hedonic Control of Eating.” Neuron 36, no. 2: 199–211. 10.1016/S0896-6273(02)00969-8. 12383777

[fsn370383-bib-0035] Sarıtepeci, M. 2021. “Multiple Screen Addiction Scale: Validity and Reliability Study.” Öğretim Teknolojisi Ve Hayat Boyu Öğrenme Dergisi ‐ Instructional Technology and Lifelong Learning 2, no. 1: 1–17. 10.52911/itall.796758.

[fsn370383-bib-0036] Shambare, R. , R. Rugimbana , and T. Zhowa . 2012. “Are Mobile Phones the 21st Century Addiction?” African Journal of Business Management 6, no. 2: 573–577. 10.5897/AJBM11.1940.

[fsn370383-bib-0037] Throuvala, M. A. , M. D. Griffiths , M. Rennoldson , and D. J. Kuss . 2020. “The Role of Recreational Online Activities in School‐Based Screen Time Sedentary Behaviour Interventions for Adolescents: A Systematic and Critical Literature Review.” International Journal of Mental Health and Addiction 19, no. 4: 1065–1115. 10.1007/S11469-019-00213-Y.

[fsn370383-bib-0038] Tokunaga, R. S. , and S. A. Rains . 2010. “An Evaluation of Two Characterizations of the Relationships Between Problematic Internet Use, Time Spent Using the Internet, and Psychosocial Problems.” Human Communication Research 36, no. 4: 512–545. 10.1111/j.1468-2958.2010.01386.x.

[fsn370383-bib-0039] Wansink, B. 2010. Mindless Eating: Why we Eat More Than we Think. Bantam Books.

